# Effect of R&D activity on level of innovation of EU countries in 2014 and 2020

**DOI:** 10.1371/journal.pone.0299697

**Published:** 2024-02-29

**Authors:** Katarzyna Piotrowska, Aleksandra Szymura, Piotr Wanicki

**Affiliations:** 1 Department of Accounting, Reporting and Financial Analysis, Wroclaw University of Economics and Business, Wroclaw, Poland; 2 Department of Econometrics and Operational Research, Wroclaw University of Economics and Business, Wroclaw, Poland; Second Xiangya Hospital, Central South University, CHINA

## Abstract

Research and development carried out by companies are pivotal for innovative economies of countries, especially in the unpredictable and changing social, economic and political environment. In this context, it is very important to answer, which factors identify the effectiveness of measures in relation to R&D activity and innovativeness in EU countries and how should the degree of development of a country be assessed in terms of R&D activity? The purpose of this article is to verify level of innovativeness and degree of research and development (R&D) activity in EU countries in the years 2014 and 2020 using Hellwig’s measure of development. To achieve this, qualitative and quantitative analysis, synthesis, deduction and induction, comparative analysis, and reasoning by analogy of phenomena were employed. The research was conducted on the basis of the expertly selected variables for their relation to R&D activity from a number of sources, such as Eurostat, World Bank Data, etc.. The indicated variables were analysed using statistical methods and then subjected to a linear ordering procedure based on the Hellwig development pattern method. Thanks to the research results, it is possible to indicate areas in which the initiation of activities would have the greatest degree of influence on development of R&D activity, thus influencing the increase in the level of innovativeness of a country. Indicators relating to R&D activity were selected and then used as variables to study the effect of the degree of R&D activity in EU countries in the years 2014 and 2020 on the level of innovativeness of these countries. The conducted research coincides with the results presented in the European Innovation Scoreboard. There is a significant correlation between the development of R&D activities and innovation performance.

## Introduction

### Background

A key factor in the innovativeness of the economies of individual countries is research and development activity, especially in an unpredictable and changing socio-economic and political environment. For many years, Poland has intensified activity in the field of development, thanks to which Poland’s GDP has increased at a stable and rapid rate, reducing the differences with other EU countries, faster even than the other countries in the Visegrad Group. Nevertheless, the level of investment in research and development in Poland is still at a low level, resulting in a low level of innovativeness in the national economy as a whole. Although the share of expenditure on research and development activity in relation to GDP is growing, it has not reached the planned level for this indicator (the indicator reached a level of only 1.39% in 2020 [1], despite a planned level of 1.7% [2]). This means that it is necessary to continue supporting businesses by eliminating barriers to the undertaking of research and development activity, or by proposing new more effective solutions in selected fields of activity (social, economic and political).

In this context, there are key doubts as to the answers to the questions: Which factors influence the effectiveness of measures in relation to R&D activity in EU countries? How should the degree of development of a country be assessed in terms of R&D activity, the principal driving force behind the innovativeness of economies? To answer such questions, the authors undertook research, the aim of which is to verify the influence of the degree of research and development (R&D) activity in EU countries in the years 2014 and 2020 on their level of innovativeness. To achieve this, it was necessary to select factors that had the greatest influence on R&D activity in EU countries in the years 2014 and 2020. The research proposes a synthetic measure, thanks to which a ranking of EU countries was prepared for the years 2014 and 2020 that enabled comparison of the countries according to selected areas of R&D activity. The adopted 2014–2020 period coincides with the financial perspective of European Union programs. Members of the European Union during this period were able to use instruments to support innovation. This approach is well established in the literature [3–6]. The research used the linear ordering procedure based on the method of Hellwig’s development pattern. The selection of variables required the use of a Spearman’s rank correlation coefficient, which by definition is more resistant to the occurrence of outlying observations. In order to select the most appropriate variables for the research in terms of the illustration of R&D activity against the innovativeness of individual countries, a broader scope of such variables was determined, which was subject to selection.

The research process was planned in detailed according to the following stages:

1. The analysis was conducted of the published indicators, which were then subject to selection in order to choose the most representative within the indicated environment for R&D activity, assuming that this stage of the process was of key importance for innovativeness.
2. The research used the linear ordering procedure based on the method of Hellwig’s development pattern. The research procedure was shown to be successful in research by E. Roszkowska and M. Filipowicz-Chomko [7], and was verified in research by the authors [8]. The selected indicators were tested according to their statistical characteristics and accepted as variables in the procedure of indicating a synthetic measure of innovativeness according to the level of R&D activity in these countries.
3. The research classified EU countries into four groups according to the level of the measure achieved. Final conclusions were formulated on the basis of the research results.

Achievement of planned research aim required the use of suitable research methods:

- qualitative analysis (observation of economic practices with the use of earlier research by the authors, including survey research),
- synthesis, induction and deduction (analysis was conducted of selected variables on the basis of the areas identified, with variable selection made with the use of the Spearman’s rank correlation coefficient),
- analogy (understanding through similarities–comparative analysis),
- reasoning (conclusions drawn with the use of the linear ordering procedure based on the method of Hellwig’s development pattern).

The article follows a classical structure, where it begins with a literature review of innovation research, followed by the presentation of the study’s methodology, and the subsequent results on the impact of R&D on the innovation of EU countries. Finally, a discussion is held and the conclusions of the study are presented.

## Literature review

Being subject to continuous changes in the environment and strong market competition (national, European and global), firms are forced to conduct their activity in a flexible manner. This means skilful adaptation of their activity to the needs and expectations of the market through the continuous implementation of changes, thanks to which enterprises can achieve and maintain a competitive advantage. These activities must be innovative in nature, which involves the successful use of new ideas in practice, and the application of specific tools that the enterprise undergoing change uses as an opportunity for a different business activity or service [9]. It can even be said that innovation is a process, as a result of which every material or non-material change allows a firm to operate smoothly on the market and achieve better economic results. Both internal factors inside the company and external factors [10] can be the inspiration for innovation, and should be taken into account in the innovation management process, with selection of the appropriate effort and resources and their correct distribution and coordination. Successful innovation therefore depends on many factors (employees, equipment, knowledge, financial resources etc.) [9], as well as skills for the conducting of research and development activity. Innovative businesses create innovative economies, and have a direct influence on the level of innovativeness of individual countries. Measuring the innovativeness of countries reveals which are performing the best in this respect [11].

The innovativeness of businesses was studied earlier in the previous century. J.A. Schumpeter [12] developed a model for the occurrence of innovation, and was considered to be the precursor of the theory in this respect, stating that “innovation is the implementation of a new or improved existing product, the introduction of a new method of production, the acquisition of new sources and possibilities, the use of areas and market as yet untouched, and new ways of organising business” [12, 13]. Innovation is therefore a tool for entrepreneurship and allows resources to be used to create new wealth, as well as enabling the creation of a new product or the discovery of a new use for a specific object [14]. The idea behind innovation is the deliberate, organised quest for changes, and the systematic analysis of opportunities for social or economic innovation that would enable such a change [15]. Understood in this way, innovation can take the form of a project, invention, service, process, system, organizational structure or business model. Meanwhile, the innovation process involves “the deliberate implementation of changes by individuals (…), based on replacing the current state of affairs with another, additionally assessed in the light of defined criteria, and coming together to comprise progress” [16]. Innovation is therefore a sequential set of actions consisting of the creative preparation and shaping of a blueprint for a new state of affairs that satisfies a defined human need, which is then put into practice in a certain fragment of one of the elements of the ‘global system’ [17].

For many years there have been multiple definitions and classifications of the innovations implemented by enterprises [15], these have indicated the specific attributes of innovations, [18] their type, phases of implementation, risk [19, 20] and way they are managed [9], to the open innovation paradigm [21], which has garnered increasing importance in academic research and industrial applications [22]. Design and innovation was consider as two of the central elements necessary for the long term success of business in the manufacturing sector already in the 1970s [23]. In turn Unger and Zagler [24] analyzed four prototypic models of innovation, from a simple technological model to an elaborated institutional model that included financial, organizational and technological variables [24], also public policy has an important role to play in promoting research and development (R&D), diffusion, and use of new knowledge and innovations. Fiscal incentives, including tax policies, should be directed at specific barriers, impediments or synergies to facilitate the desired level of investment in R&D and innovations, as Appelt, Bajgar, Criscuolo, in, [25] claim. Should be noted that excellent research may contribute also to successful science based technological innovation [26].

In every case, it was underlined that the research and development process of a given undertaking was of key importance for the emergence of innovation, as there is no innovation without research and development work. Thus, there are definitions of these categories in the theory and practice of management sciences, economics and finance [27–29]. In every case their innovative character is underlined depending on whether they consist of research work with an unpredictable end result, or are developmental for which ultimate results can be identified [27]. In economic practice, this activity is also called research and development (R&D). For the purposes of this research, R&D activity is defined as the development or improvement of existing products and processes, including the creation and design of new, modified or improved products and processes, and the development of experimental projects and prototypes [1].

A systematic review was conducted, in accordance with the accepted procedure study [30, 31] using the VOSviewer method [32] and an integrative review of the widely applied and most recent systematic literature reviews guidelines in the management domain [33], in order to indicate the current state of knowledge on the research in terms of innovativeness and the importance of research and development work in the innovation process. The Scopus database was used for this purpose. In the first step, the keywords were defined on the basis of the authors’ experience, as well as on example literature in this field. On this basis, an enquiry was prepared for the Scopus database: TITLE-ABS-KEY("R&D indicator" or "R&D indicators" or "R&D system" or "R&D performance" or "R&D capabilities" or "R&D determinant" or "R&D determinants" or "R&D factor" or "R&D factors" or "competitiveness" or "R&D performance") AND ("comparative analysis" or "multicriteria analysis" or "rank" or "ranking" or "model" or "models" or "tool" or "tools" or "Hellwig method" or "method" or "methods" or "mapped" or "mapping" or “report” or “index”) AND (“innovation”) AND LANGUAGE("English") AND DOCTYPE("ar") AND SRCTYPE("j").

**Fig 1:**
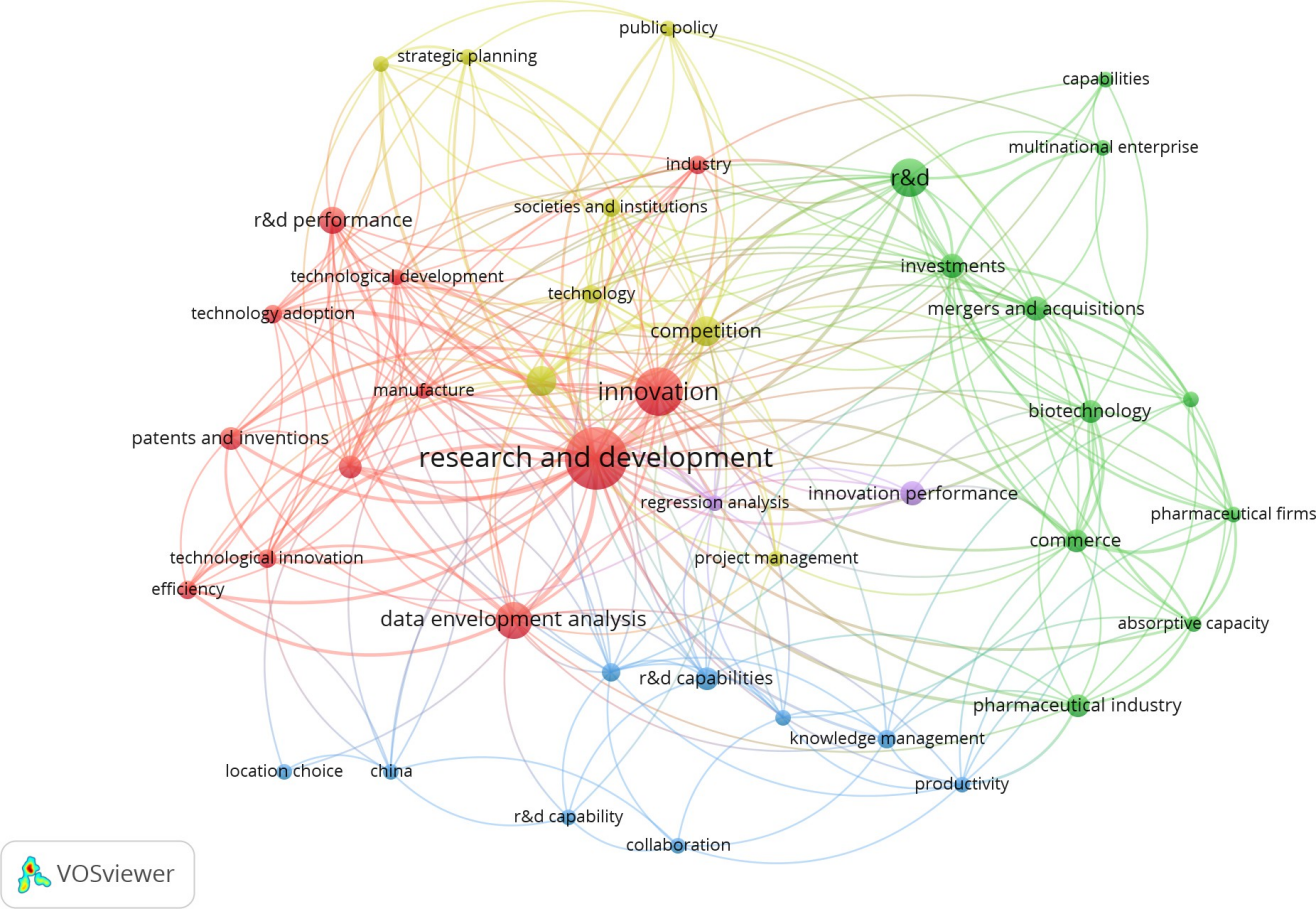
Bibliometric map visualization. Own elaboration using the VOSviewer tool.

The enquiry limited the search to articles in English published in journals. This search returned a total of 595 results. Selection was then made of the publications on the basis of an ABS list (it was accepted that articles from journals with a score of 3 or higher would be included in further analysis). After this criterium was applied, 154 publications remained for analysis. The abstracts of these articles were then read, and on the basis of expert knowledge, selection was made of those that were connected with the topic of innovativeness and research and development work. This analysis resulted in the selection of 114 publications, which were used to generate a bibliometric visualisation map using the VOSviewer tool, as presented in Fig 1.

On the basis of the keywords identified by VOSviewer, these were divided into clusters (it was assumed that a given keyword must appear in at least 3 articles). The division of the keywords into clusters is presented in Table 1.

**Table 1:** Key characteristics divided into clusters.

No.	Cluster	Key characteristics
1.	red	Data envelopment analysis, efficiency, industrial performance, industry, innovation, manufacture, patents and inventions, R&D performance, research and development, technological development, technological innovation, technology adoption
2.	green	Absorptive capacity, biotechnology, capabilities, commerce, investments, mergers and acquisition, multinational enterprises, pharmaceutical firms, pharmaceutical industry, R&D, technology transfer
3.	blue	China, collaboration, firm performance, knowledge management, location choice, performance, productivity, R&D capabilities, R&D capability
4.	yellow	Competition, project management, public policy, research and development management, societies and institutions, strategic planning, technological forecasting, technology
5.	purple	Innovation performance, regression analysis

Own elaboration using the VOSviewer tool

The identified clusters define the various areas of research presented in the analysed literature. The first (and largest) cluster contains keywords related to the effectiveness of research and development work, and technological aspects of the emergence of innovation. The next cluster relates to commercialisation, the transfer of technology and investment in R&D. The blue cluster includes research into the management of knowledge, productivity and cooperation in the field of R&D. The yellow area relates to project and technology management as well as strategic planning. The last area (purple) includes key terminology related to regression analysis in the field of innovativeness.

**Fig 2:**
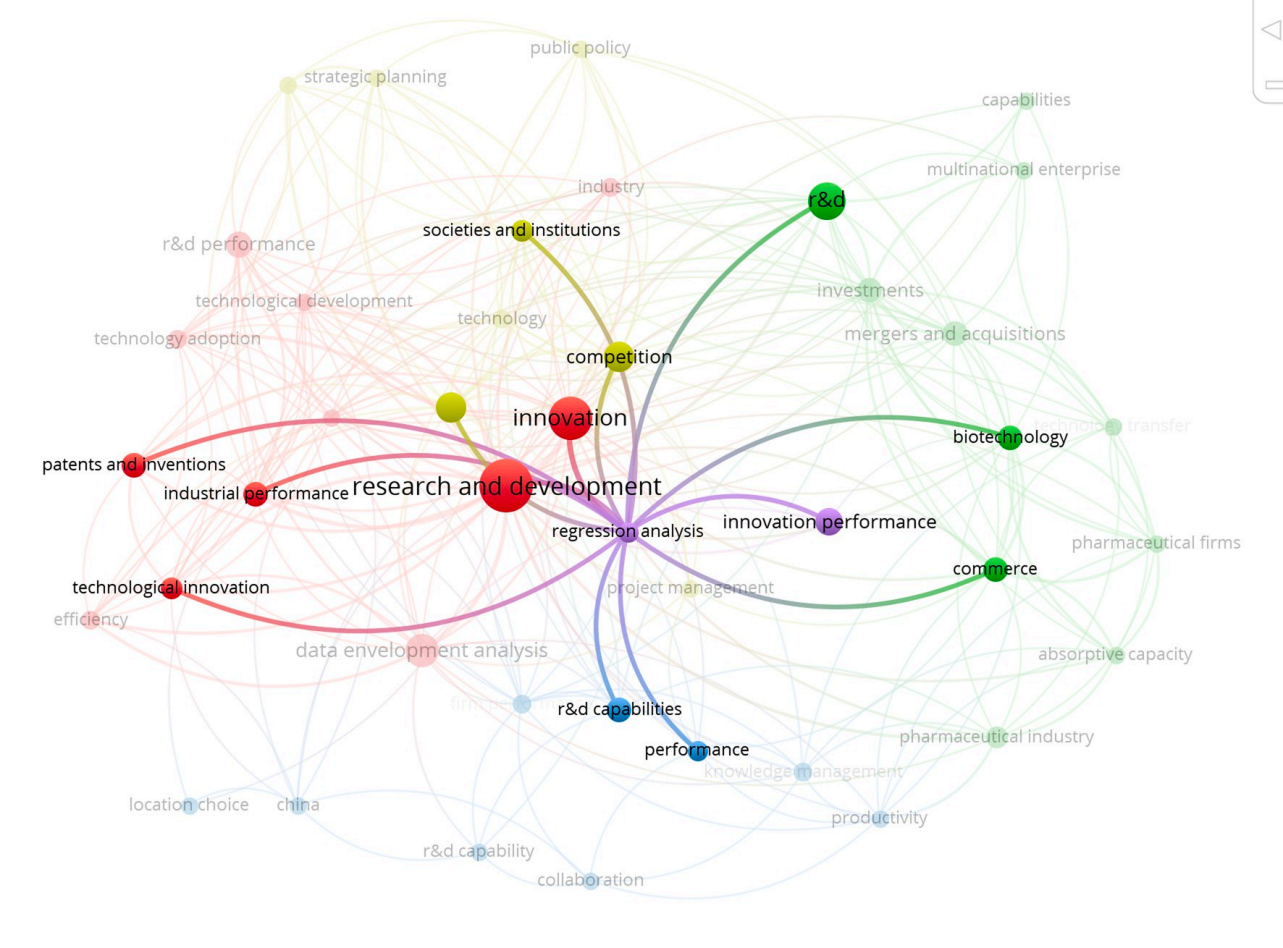
Results of the analysis of connections between words from selected clusters. Own elaboration using the VOSviewer tool.

**Fig 3:**
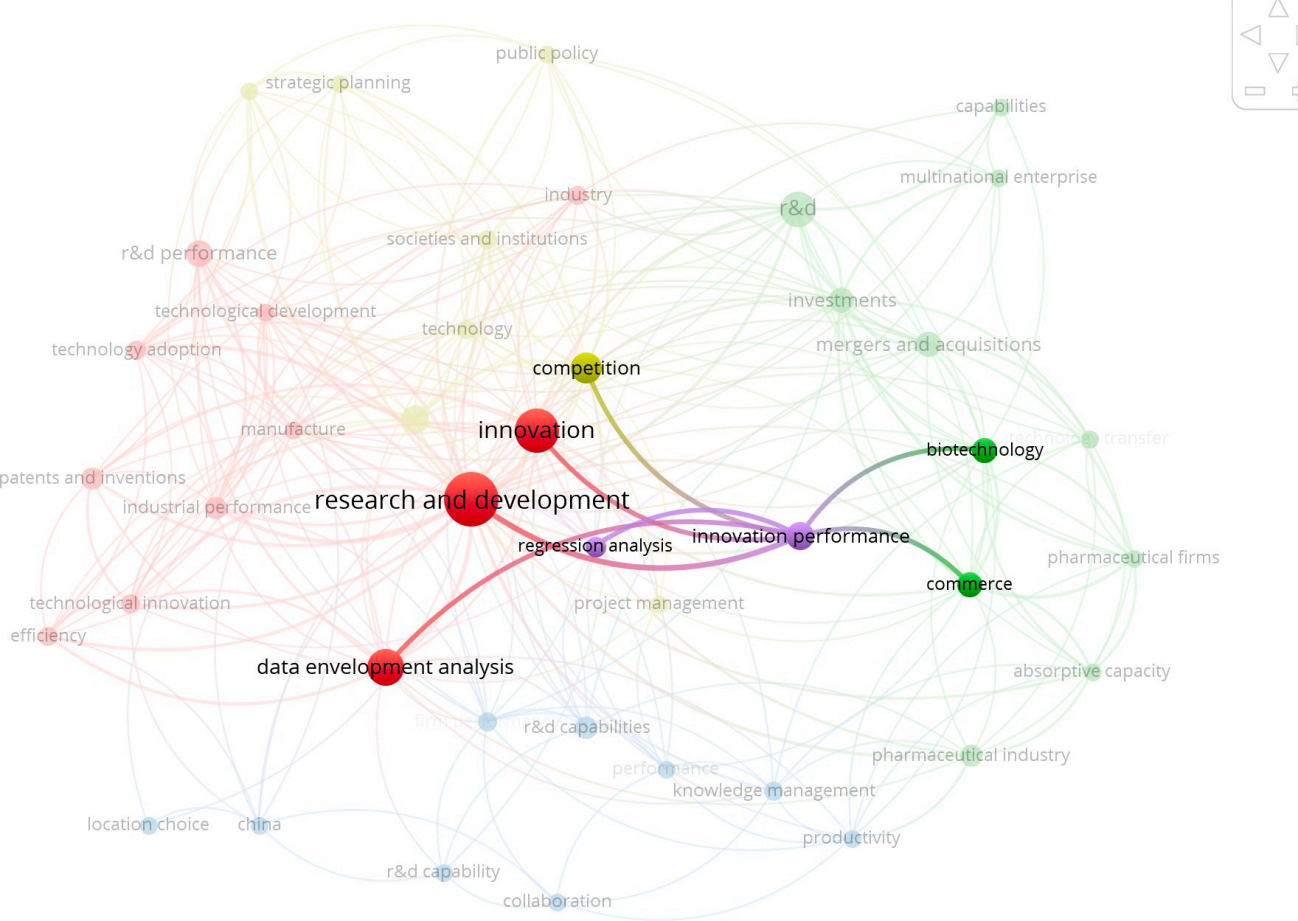
Results of the analysis of connections between words from selected clusters. Own elaboration using the VOSviewer tool.

On the basis of the bibliometric map, it can be said that the area related to analysis of factors that have an influence on the level of innovativeness of a given country/region is very low. Among the keywords in an area related to regression analysis, however it is very small. Studies have been reported in the literature on measuring innovation [34–36], the dimensions of measuring innovation and R&D performance [37, 38], to select various indicators and methods for its assessment [8]; the literature review in term of innovativeness measures were classified in terms of inputs, capabilities and outputs [39], impact of expenditure on research and development activity on its results [40], the relationship between innovation investment and regional innovation performance [41], the use of innovation scoreboards as a main device for policy monitoring and benchmarking [42], a procedure of measurement of innovativeness growth over time [43], taxonomic methods of measure development in this innovation area [44] and formal model for measuring R&D performance [45]. On this basis, the variables presented in the research methodology were selected. Analysis was also conducted of the connections between words from the purple cluster and other keywords in other clusters, the results of which are presented in Figs 2 and 3.

## Research methods

Achieving the research aim required the implementation of specific research stages, beginning with indication of the variables, and subjecting them to analysis in order to remove those that were not comparable for the two periods (2014 and 2020). The variables were then tested using the Spearman’s rank correlation coefficient so as to select the most important measures of innovativeness, and were then subject to a linear ordering procedure based on the method of Hellwig’s measure of development.

The study compared countries that were members of the European Union at the end of 2020. The United Kingdom, which left the European Union at the beginning of 2020, was not included.

### Stage 1: Analysis of variables

In the first stage, 19 variables were selected. The specifics of the variables and their source (Access: 30.10.2022) of origin are presented in Table 2. The variables were selected based on an expert analysis of publicly available statistics related to innovation and R&D, as well as an analysis of the literature in this area [46, 47].

**Table 2:** Characteristics of the variables and their source of origin.

Abbreviation	Variable description	Source
Share_R&D	Share of R&D personnel and researchers in total active population and employment by sector of performance and gender	Eurostat
GERD_per_inhabitant	GERD by sector of performance and type of R&D as euros per inhabitant	Eurostat
Share_GERD	GERD by sector of performance and type of R&D as percentage of GDP	Eurostat
Citations	Citations per document	SJR ‐ International Science Ranking
Share_GBARD	Share of GBARD in total general government expenditure	Eurostat
Int_scientific	International scientific co-publications per million population	European Innovation Scoreboard Database
R&D_exp_public	R&D expenditure in the public sector as percentage of GDP	European Innovation Scoreboard Database
Ven_cap_exp	Venture capital expenditure as percentage of GDP	European Innovation Scoreboard Database
Med_High_prod_export	Medium and high technology product exports	European Innovation Scoreboard Database
Knowledge_export	Knowledge-intensive services exports as percentage of total services exports	European Innovation Scoreboard Database
Doctorates	New graduates at doctoral level in science, mathematics, computing, engineering, manufacturing and construction per 1000 of the population aged 25–34	Eurostat
Gov_Index	E-Government Index	UN E-Government Knowledgebase
Part_Index	E-Participation Index	UN E-Government Knowledgebase
Online_Serv_Index	Online Service Index	UN E-Government Knowledgebase
Human_Cap_Index	Human Capital Index	UN E-Government Knowledgebase
Tel_Inf_Index	Telecommunication Infrastructure Index	UN E-Government Knowledgebase
Patent_applications	Patent applications to the European Patent Office by applicants’/inventors’ country of residence per million inhabitants	Eurostat
Researchers_R&D	Researchers in R&D per million people	World Bank Data
Business_start	Starting a business–Score	World Bank Data

Own elaboration

All the gathered variables were subject to initial analysis taking into account basic statistics. Table 3 present basic statistics for 2014 and 2020 for the variables indicated earlier.

**Table 3:** Statistics used in the research for 2014 and 2020 for the indicated variables.

Variable	Missing values		Mean		Standard deviation		Coefficient of variation [%]		Skewness coefficient	
	2014	2020	2014	2020	2014	2020	2014	2020	2014	2020
Share_R&D	0	0	1.12	1.32	0.51	0.48	45.66	36.66	0.33	-0.16
GERD_per_inhabitant	0	0	495.31	608.32	458.05	513.13	92.48	84.35	0.80	0.74
Share_GERD	0	0	1.60	1.77	0.87	0.89	54.31	50.17	0.55	0.58
Citations	0	0	21.18	6.06	5.75	1.40	27.15	23.14	-0.06	0.54
Share_GBARD	0	0	1.14	1.17	0.44	0.41	39.07	35.29	0.20	0.19
Int_scientific	0	0	961.42	1389.27	581.20	786.44	60.45	56.61	0.56	0.57
R&D_exp_public	0	0	0.62	0.64	0.23	0.26	37.68	40.49	0.20	0.14
Ven_cap_exp	0	0	0.07	0.13	0.04	0.09	64.19	74.11	0.71	0.72
Med_High_prod_export	0	0	48.35	52.02	12.41	11.16	25.66	21.46	-0.32	-0.19
Knowledge_export	0	0	53.00	62.86	19.89	18.54	37.52	29.50	0.33	-0.02
Doctorates	1	1	0.74	0.64	0.37	0.28	49.87	43.46	0.35	0.01
Gov_Index	0	0	0.72	0.85	0.10	0.06	13.50	6.81	-0.08	0.49
Part_Index	0	0	0.60	0.83	0.19	0.11	31.75	13.19	0.00	-0.36
Online_Serv_Index	0	0	0.63	0.82	0.18	0.10	28.38	12.25	0.01	-0.16
Human_Cap_Index	0	0	0.86	0.89	0.05	0.05	5.52	5.47	-0.07	-0.22
Tel_Inf_Index	0	0	0.68	0.84	0.12	0.07	17.82	8.17	0.10	0.39
Patent_applications	0	0	142.48	144.20	191.06	167.81	134.10	116.37	1.92	1.33
Researchers_R&D	0	0	3537.30	4249.77	1747.38	1793.37	49.40	42.20	0.59	0.33
Business_start	0	0	87.37	89.71	5.59	4.25	6.39	4.74	-0.70	-0.31

Own elaboration based on statistical data

The main assumption of the research was consideration in the years studied (2014 and 2020) of the same variables, which is why in the first place, variables were removed which in at least one of the studied periods did not fulfil one of two conditions, that is:

- the number of cases of missing data was 0,
- the value of the coefficient of variation was higher than 10%.

The variables removed have been highlighted in Table 3, and a list of the variables with the reason for elimination is given in Table 4.

**Table 4:** List of variables not used in the research.

Variable	Reason for elimination
Doctorates	Missing values in 2014 and 2020
Gov_Index	Coefficient of variation in 2020 < 10%
Human_Cap_Index	Coefficient of variation in 2014 and 2020 < 10%
Tel_Inf_Index	Coefficient of variation in 2020 < 10%
Business_start	Coefficient of variation in 2014 and 2020 < 10%

Own elaboration

### Stage 2: Correlation analysis

On the basis of this selection procedure, 14 variables were taken into consideration in further analysis. Analysis was conducted of the correlation between indicated variables. To achieve this, the Spearman’s rank correlation coefficient was used, which is by definition more resistant to the occurrence of outlying observations than, for example, Pearson’s correlation coefficient. In the literature [48, 49], strong dependence between variables occurred when the values of the correlation coefficient were higher than 0.7 in absolute value–the value of the Spearman’s rank correlation coefficient was lower than −0.7 and greater than 0.7. To begin with, the number of variables correlated with a given variable at the accepted level was tested for both years. If more than one variable had the same number of correlated variables, and these were in addition correlated with one another, the variable with the highest coefficient of variation was selected for further stages. The remaining variables were removed from further analysis. If at least two variables had the same number of variables correlated with them, and they were not correlated with one another at the accepted level, all of them were removed. For the final considerations, variables were chosen that were identified in the correlation analysis for both 2014 and 2020. The individual stages of eliminating variables for 2014 are presented in the next steps:

- Step I: the variable GERD_per_inhabitant, which is correlated with the greatest number of variables (8), was removed,
- Step II: the variables Share_R&D, Share_GERD and Researchers_R&D are correlated with the greatest number of variables (6) and strongly correlated with one another. For further analysis, Share_GERD due to it having the highest coefficient of variation value among these variables was taken. The variables Share_R&D and Researchers_R&D were removed,
- Step III: the variable Share_GERD, which is correlated with the greatest number of variables (4), was removed,
- Step IV: the variable Citations, which is correlated with the greatest number of variables (3), was removed,
- Step V: in the last step there were 3 pairs of variables correlated with one another. From each of these pairs, the variable with the higher coefficient of variation value was chosen for further analysis. Of these, Share_GBARD, Patent_applications and Part_Index were selected for further analysis, while R&D_exp_public, Int_scientific and Online_Serv_Index were removed,
- Step VI: none of the variables are now correlated at the accepted level, and a correlation matrix was obtained (Table 5).

**Table 5:** Correlation matrix for 2014.

Variable	Share_GBARD	Ven_cap_exp	Med_High_prod_export	Knowledge_export	Part_Index	Patent_applications
**Share_GBARD**	1.00	0.34	0.02	0.39	0.30	0.61
**Ven_cap_exp**	0.34	1.00	-0.28	0.44	0.41	0.41
**Med_High_prod_export**	0.02	-0.28	1.00	0.09	-0.29	0.27
**Knowledge_export**	0.39	0.44	0.09	1.00	0.30	0.68
**Part_Index**	0.30	0.41	-0.29	0.30	1.00	0.26
**Patent_applications**	0.61	0.41	0.27	0.68	0.26	1.00

Own elaboration

For 2020, the research procedure was conducted analogically as for the year 2014, with the individual stages of elimination of variables for 2020 as follows:

- Step I: the variable GERD_per_inhabitant, which is correlated with the greatest number of variables (7), was removed,
- Step II: as many as 5 variables are correlated with the greatest number of features (4). Among them, 4 are strongly correlated with one another (Share_R&D, Share_GERD, R&D_exp_public, Researchers_R&D), and for this reason it was decided to select the one with the highest coefficient of variation value: Share_GERD. For further stages, the variable Patent_applications was also selected, which was correlated in total with as many as 5 variables, but only with two of the previously mentioned four variables. The variables Share_R&D, R&D_exp_public and Researchers_R&D were removed,
- Step III: the variables Citations, Int_scientific and Patent_applications are correlated with one another. For further analysis, Patent_applications was selected due to it having the highest coefficient of variation value (116.37%). the remaining two, that is: Citations and Int_scientific, were removed,
- Step IV: in the last step there were 2 pairs of variables correlated with one another. From each of these pairs, the variable with the higher coefficient of variation value was chosen for further analysis. Of these, Share_GERD and Part_Index were selected for further analysis, while Share_GBARD and Online_Serv_Index were removed,
- Step V: none of the variables are now correlated at the accepted level, and a correlation matrix was obtained, the results of which are presented in Table 6.

**Table 6:** Correlation matrix for 2020.

Variable	Share_GERD	Ven_cap_exp	Med_High_prod_export	Knowledge_export	Part_Index	Patent_applications
**Share_GERD**	1.00	0.31	0.17	0.22	0.23	0.61
**Ven_cap_exp**	0.31	1.00	-0.11	0.66	0.28	0.56
**Med_High_prod_export**	0.17	-0.11	1.00	0.01	-0.10	0.16
**Knowledge_export**	0.22	0.66	0.01	1.00	0.11	0.63
**Part_Index**	0.23	0.28	-0.10	0.11	1.00	0.22
**Patent_applications**	0.61	0.56	0.16	0.63	0.22	1.00

Own elaboration

For the purposes of comparison, the results of correlation analysis for both years (2014 and 2020) are presented in Table 7.

**Table 7:** Results of correlation analysis for 2014 and 2020.

2014	2020
Share_GBARD	Share_GERD
Ven_cap_exp	Ven_cap_exp
Med_High_prod_export	Med_High_prod_export
Knowledge_export	Knowledge_export
Part_Index	Part_Index
Patent_applications	Patent_applications

Own elaboration

To achieve the research aim, it was necessary to accept only those variables whose selection would not be discretionary and whose correct indication would result from the correlation analysis, enabling the selection of the most representative indicators. The variables accepted for final analysis were those identified in both of the years studied, that is:

- Ven_cap_exp
- Med_High_prod_export
- Knowledge_export
- Part_Index
- Patent_applications

### Stage 3: Hellwig’s measure of development

Prior to commencing the creation of a synthetic measure of development for individual countries in the years studied, every variable was standardised according to the formula:

$$z_{ij}=\frac{x_{ij}-\bar{x_{j}}}{s_{j}},$$ (1)

wherein:

*x*_*ij*_−value of the *j* variable for the *i* object,

$\bar{x_{J}}$ –arithmetic mean of the *j* variable,

*s*_*j*_−standard deviation of the *j* variable.

In the next stage of the research, every variable was assigned information whether it was a stimulant (the higher values, the better), or a destimulant (the lower, the better). It turned out that all of them are stimulants, and therefore no additional transformations were made in order to calculate the synthetic measure of development.

**Fig 4:**
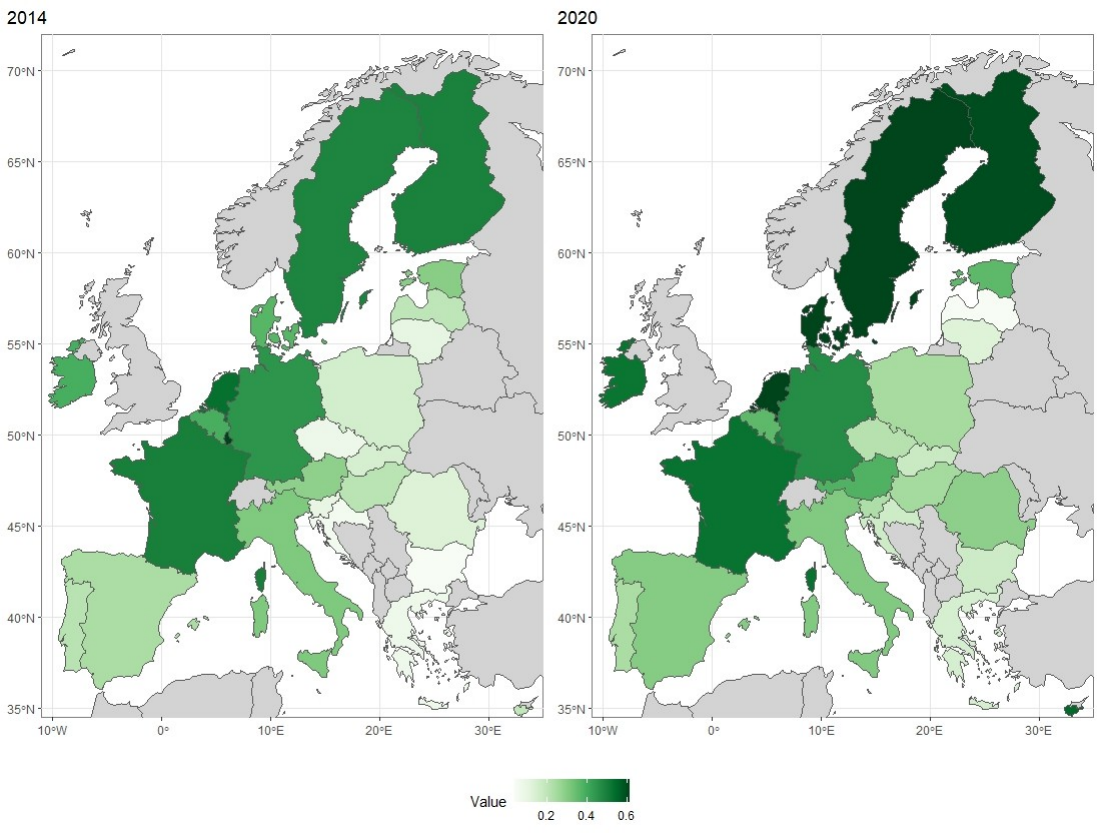
Map of EU countries according to Hellwig’s measure of development for 2014 and 2020. Own elaboration using R language.

By taking into consideration the indicated variables for each EU country, the development measure values were calculated using the linear ordering procedure based on the method of Hellwig’s measure of development [50–55]. The calculations were carried out both for 2014 and 2020, with the detailed results presented in the graph in Fig 4. All the geographic maps in this article were prepared using the R language with the packages: *rnaturalearth* [56] and *rnaturalearthdata* [57]. Both packages used as a source information from public domain Natural Earth [58].

In the first stage of the Hellwig method, the pattern objects of each variable were determined, i.e. the maximum values of the variables labelled as stimulants and the minimum values in the case of destimulants were selected. Based on these, the distance of each object from the assumed development pattern object was calculated according to the formula:

$$d_{i0}=\sqrt{\sum_{j=1}^{p}{\left(z_{ij}-z_{0j}\right)}^{2}},$$ (2)

wherein:

*z*_*ij*_−standardized value of the *j* variable for the *i* object,

*z*_0*j*_ –pattern object value of the *j* variable.

The final result, the value of the synthetic development measure, was obtained after applying the formula:

$$d_{i}=1-\frac{d_{i0}}{d_{0}},$$ (3)

wherein:

*d*_*i*0_ –Euclidean distance of *i* object from pattern object,

*d*_0_ –value calculated by the formula:

$$d_{0}={\bar{d}}_{0}+2S_{d}$$ (4)

wherein ${\bar{d}}_{0}$_,_ is the arithmetic mean, and *S*_*d*_, is the standard deviation of the distances of the objects from the pattern object.

## Results

**Fig 5:**
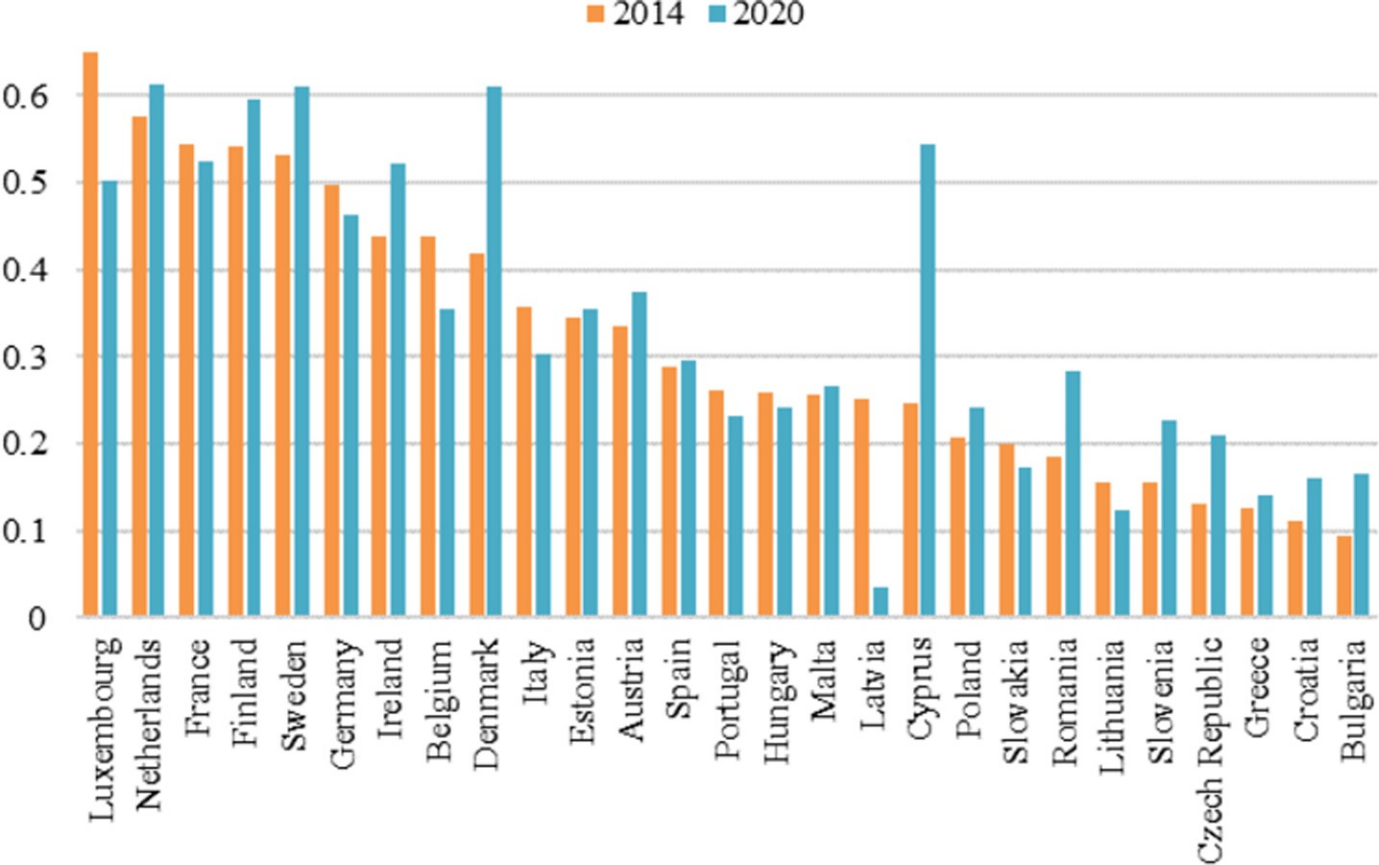
Measure values according to Hellwig’s measure of development for 2014 and 2020 –ranking. Own elaboration.

The results are presented below in graphical form (Fig 5) to enable the comparison of countries with similar measure values.

The measure values for individual countries were presented in Fig 4. and additionally presented in graphical form (Fig 5). The darker the colour on the map (Fig 4), the higher the value of the calculated index. The grey colour indicates countries that are not included in the study. Darker shades were observed in both years in the countries of Western Europe and Scandinavia. Lighter shades dominate in Central and Eastern European countries. Taking into account the measure values for individual countries presented, additionally, for each year, the countries were grouped according to quartile so as to enable the indication of groups of countries for each year:

- **Group I**: (quartile III; maximum value>
- **Group II**: (median; quartile III>
- **Group III**: (quartile I; median>
- **Group IV**: <minimum value; quartile I>

**Fig 6:**
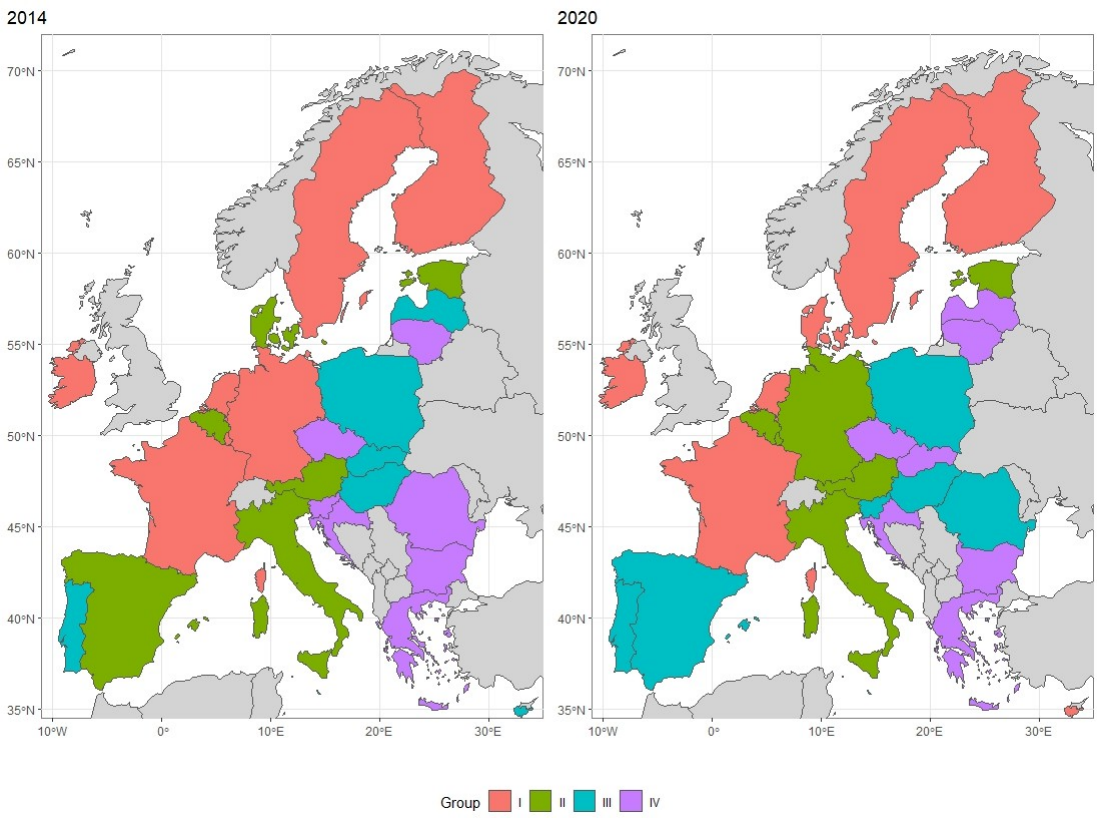
Map of EU countries according to the ranking, divided into 4 groups of similarities, 2014 and 2020. Own elaboration using R language.

On the basis of the research, a ranking was developed of countries according to measure of development values. The final method for dividing up the countries by assigning them to an appropriate group according to quartiles for the years 2014 and 2020 is presented in the Table 8 (within each group, the countries are sorted according to the measure of development value–from the highest to the lowest). The applied division of the results is presented below in the form of a map (Fig 6).

**Table 8:** Ranking of countries according to measure of development with division into groups of similarities.

Group I		Group II		Group III		Group IV	
2014	2020	2014	2020	2014	2020	2014	2020
Luxembourg	Netherlands	Belgium	Luxembourg	Portugal	Spain	Romania	Czech Republic
Netherlands	Sweden	Denmark	Germany	Hungary	Romania	Lithuania	Slovakia
France	Denmark	Italy	Austria	Malta	Malta	Slovenia	Bulgaria
Finland	Finland	Estonia	Belgium	Latvia	Hungary	Czech Republic	Croatia
Sweden	Cyprus	Austria	Estonia	Cyprus	Poland	Greece	Greece
Germany	France	Spain	Italy	Poland	Portugal	Croatia	Lithuania
Ireland	Ireland			Slovakia	Slovenia	Bulgaria	Latvia

Own elaboration

The measure of development results calculated for 2014 and 2020 were compared, and the calculations indicating a difference in measure of development values between the years 2020 and 2014 were calculated according to the formula:

$$difference=MR_{2020}-MR_{2014}$$ (5)

**Fig 7:**
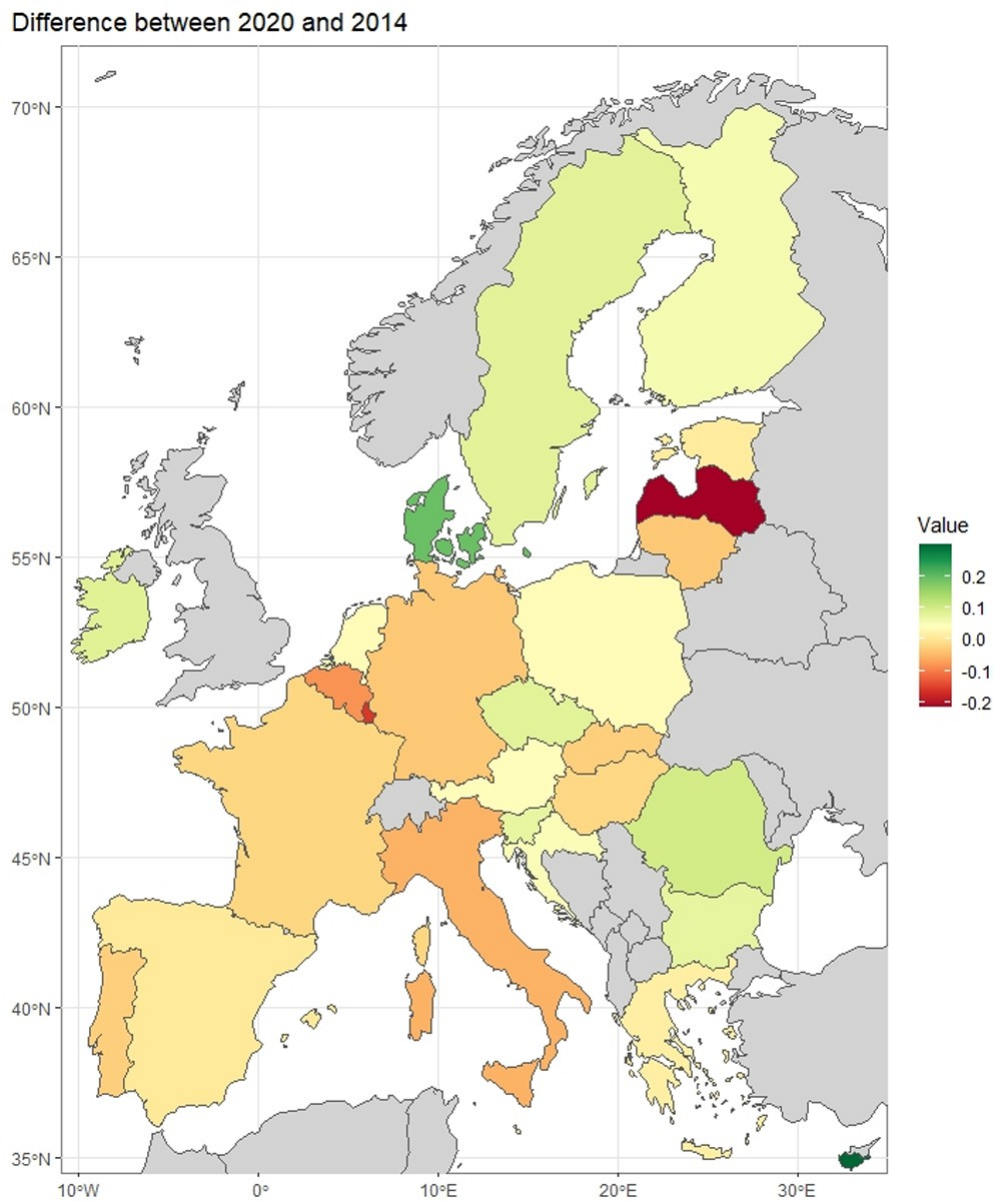
Measures of development for EU countries in the years 2014 and 2020. Own elaboration using R language.

**Fig 8:**
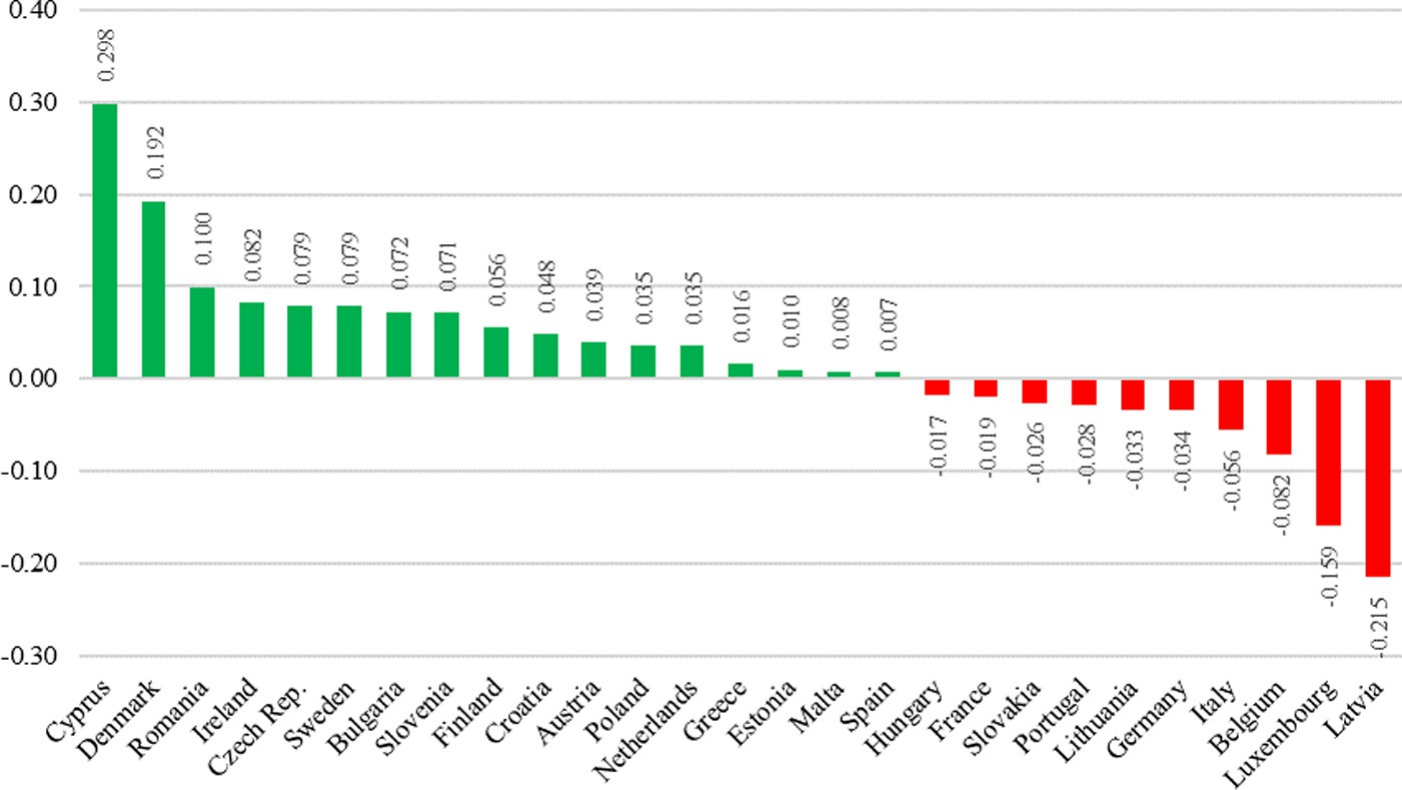
Difference in measure of development index between the years 2020 and 2014. Own elaboration.

The final research results are presented in graphical form the use of a map (Fig 7) and a bar chart (Fig 8), enabling discussion, the formulation of final conclusions and indication of further research directions.

For most countries, the value of the index in 2020 was higher compared to 2014. Compared to 2014, nine countries have changed group membership. Four of them were promoted from the lower to the higher group, and five dropped from the higher to the lower group.

The greatest increase in the value of the development index in the years under review was observed for Cyprus. The value in 2020 increased by 0.298 compared to 2014. Cyprus moved from Group III to Group I. The second largest growth in the value of the synthetic index was noted for Denmark. In 2020, the value increased by 0.192 compared to 2014. Denmark moved from Group II to Group I. In both countries, it was due to a large increase in venture capital expenditures and E-Participation Index value in 2020 compared to 2014.

The largest decrease in the value was observed for Latvia, where the value decreased by 0.215 in 2020 compared to 2014. Latvia moved from Group III to Group IV. In 2020, Latvia was classified as the last country in the ranking. It was largely due to a decrease in the value of the E-Participation Index and venture capital expenditures in 2020 compared to 2014. Latvia dropped from the top 10 in 2014 to last place in 2020 in the ranking of countries in terms of the E-Participation Index.

A huge decrease in the value of the development index was also observed for Luxembourg, which was the top-ranked country in 2014. In 2020, the value decreased by 0.159 compared to 2014 and Luxembourg moved from Group I to Group II. Despite this, it is still among the top 10 countries in 2020. Luxembourg’s decline may be due to the fact, that in 2014, the country had a significant lead in venture capital expenditures (along with France) and the number of patent applications per million inhabitants over the other countries. In 2020, this predominance has decreased significantly. Other countries have started to catch up with Luxembourg in terms of the number of patent applications per million inhabitants, and it has even been significantly overtaken in the ranking of venture capital expenditures by Cyprus.

Countries like the Netherlands, France, Finland, Sweden, Ireland, were in each year in Group I, which means that in each year their index values were in the top 25 per cent of all values. They could be called innovation leaders in the European Union in both 2014 and 2020

In contrast, index values for Lithuania, Czech Republic, Greece, Croatia, and Bulgaria, were in the worst 25 per cent of all values in both 2014 and 2020. They could be called early innovators in the European Union in both years.

Poland was classified in Group III in each year examined, but the value of the index increased by 0.035 in 2020 compared to 2014. It ranked among the top countries in Central and Eastern Europe. In this region, Estonia stood out significantly, as the only one of the Central and Eastern Europe countries to be placed in the first or second group.

## Discussion

Based on a literature review and existing innovation rankings, it is evident that R&D activity plays a central role in determining a country’s level of innovation. In addition to R&D-related factors, innovation rankings consider various indicators associated with a country’s legal and economic conditions. Notably, R&D activities are inseparable from innovation; they form an integral part of the innovation process regardless of the specific type, direction, or goals of innovation. While innovation may have diverse manifestations, R&D activity itself is well-defined and universally applicable across different fields of knowledge and practice. This characteristic is emphasized in the initial part of our article. As a result, the study’s first stage involved a critical analysis and evaluation of innovation indicators found in statistical databases. The selection process focused on identifying indicators directly linked to R&D activity, as they significantly influence enterprise activities within the respective economies.

An attempt was made to examine which of a broad list of expertly selected variables impact the innovation performance of European Union countries. Traits with either a low coefficient of variation or missing data were excluded from the study. Subsequently, a correlation analysis was performed on the remaining variables. Any highly correlated variables were then removed from consideration. The complete procedure is detailed in the research method section.

The study focused on assessing the influence of research and development (R&D) activities’ implementation in EU countries during 2014 and 2020 on their innovation levels. To conduct the investigation, a linear ordering procedure based on Hellwig’s development pattern method was utilized. The selection of these specific periods aligned with the funding cycles of R&D activities under EU programs (2014–2020 funding period). The results of the study allowed the classification of EU countries into four distinct groups based on their level of innovation, which was determined by the degree of R&D activity implementation. Through a comparative analysis, the study provided detailed insights into countries that advanced in the rankings due to their performance and those that were grouped with countries exhibiting lower levels of innovation, attributed to the level of R&D activity implementation. Additionally, the study encompassed a correlation analysis, establishing a connection between the author’s ranking of countries using the calculated development measure and rankings based on the European Innovation Scoreboard (for 2020) [59] and the Innovation Union Scoreboard (for 2014).

The correlation between the ranking created using Hellwig’s method and the European Innovation Scoreboard Index ranking was checked. Again, the Spearman’s rank correlation coefficient was used. Due to the lack of data for the EIS ranking for 2014, calculations were only made for 2020. The value of the correlation of coefficient was 0.857. This means that there is a strong correlation between both rankings. The statistical significance of this correlation was also tested. At a significance level of 0.05, the null hypothesis was rejected, and the alternative hypothesis was accepted–correlation between both rankings was significant.

Remarkably, the top four in both rankings in 2020 included the same set of countries: Denmark, Finland, Netherlands, Sweden. The only difference was in their order. In the EIS ranking, Sweden was at the top, while in the second ranking, created by the authors of this publication, the Netherlands was at the top.

For 2014, the correlation between the ranking created using Hellwig’s method and the Innovation Union Scoreboard Index ranking was checked. Again, the Spearman’s rank correlation coefficient was used. The value of the correlation of coefficient was 0.792. This means that there is a strong correlation between both rankings. The statistical significance of this correlation was also tested. At a significance level of 0.05, the null hypothesis was rejected, and the alternative hypothesis was accepted–correlation between both rankings was significant. In the ranking created by the authors, Luxembourg was at the top, while in the IUS ranking, Sweden was at the top.

Completion of the research process required the completion of the adopted tasks according to the described steps, within each task a critical discussion was conducted, which also allowed to formulate the final conclusions.

## Conclusion

The literature and previous studies consistently highlight research and development as a primary factor influencing innovation activity. While current innovation rankings consider these factors, they are not the sole determinants in the final assessment. These rankings also incorporate other elements, such as legal conditions for conducting business and overall development factors of a country, for a final evaluation. The existing rankings typically present a country’s position or its total points based on the specified criteria. However, they do not indicate which countries are experiencing the most rapid development in terms of increasing their aggregate score or improving their position in the ranking. Against this backdrop, the research outlined in the article pursued two main objectives. Firstly, it aimed to identify the variables from the rankings that are directly associated with R&D activities and to establish a measure of R&D development for each studied country within the context of innovation. Subsequently, the obtained results were compared with the European Innovation Scoreboard (for 2020) and Innovation Union Scoreboard (for 2014).

The research findings show a high level of agreement with the existing innovation rankings. The calculated measure of development using Hellwig’s method largely aligns with the clusters identified by the Union Scoreboard Index, indicating a substantial correlation between R&D development and innovation performance. Among the European Union countries studied, Cyprus, Denmark, and Romania emerged with the highest growth in created synthetic measure of development between 2014 and 2020 years, signifying their strong commitment to innovation activities and R&D investments. The significance of this research lies in the fact that previous studies have not specifically assessed measures of development to identify which countries are experiencing the most rapid advancements in terms of innovation.

The conducted studies offer valuable insights into identifying countries with effective policies in fostering innovation levels. To further this understanding, it is crucial to analyze the conditions and instruments that support innovation policies in these countries. Based on this analysis, a comprehensive solution can be formulated to support the development of the innovation ecosystem. The findings from the studies can serve as a foundation for EU institutions to develop a tool that stimulates the innovation growth of individual countries. This tool can be designed to allocate financial resources from the EU budget strategically, promoting innovation-driven development across the member states. When devising the planned support instruments, it is essential to draw upon successful solutions that have been tested and proven effective in other countries.

However, the survey has certain limitations that should be considered. One primary limitation is the selection of variables representing R&D activities solely based on their presence in innovation rankings and expert assessment. This subjectivity may introduce bias into the study’s findings. To address this limitation, future research could undertake a more comprehensive analysis of variables influencing R&D activity, considering a broader range of indicators and data sources.

Additionally, the research relies solely on the Hellwig method for measuring development. To strengthen the study’s findings, it would be beneficial to compare the results with other linear ordering methods, such as TOPSIS. Exploring multiple methods could offer a more comprehensive perspective on the relationship between R&D development and innovation outcomes.

The article presents a novel and valuable approach to measuring the development of innovation in European Union countries, utilizing Hellwig’s measure of development, this can be taken as the practical implications. This innovative solution enables the identification of countries experiencing the most rapid development in R&D activity, a key determinant of innovation.

In the future, the use of clustering techniques, like the *k*-means algorithm or DBSCAN, could enhance the research by identifying groups of countries with similar characteristics in terms of R&D activity and innovation. Additionally, DBSCAN’s capability to detect outliers would be useful in identifying countries that significantly excel or lag behind others in terms of innovation development.
